# Effect of antenatal educational intervention on maternal breastfeeding self-efficacy and breastfeeding success: a quasi-experimental study^*^

**DOI:** 10.1590/1980-220X-REEUSP-2021-0428

**Published:** 2022-04-04

**Authors:** Rukiye Öztürk, Sibel Ergün, Nurcan Özyazıcıoğlu

**Affiliations:** 1 Bandırma Research and Training Hospital, Breastfeeding Counseling, Balıkesir, Turkey.; 2 Balıkesir University, Faculty of Health Sciences, Department of Pediatric Nursing, Balıkesir, Turkey.; 3 Bursa Uludağ University, Faculty of Health Sciences, Department of Pediatric Nursing, Bursa, Turkey.

**Keywords:** Breast Feeding, Human Milk, Lactation, Nursing Education, Prenatal Education, Aleitamento Materno, Leite Materno, Lactação, Educação em Enfermagem, Educação Pré-Natal, Lactancia Materna, Leche Humana, Lactancia, Educación Continua en Enfermería, Educación Prenatal

## Abstract

**Objective:**

To examine the effect of breastfeeding educational intervention given in the antenatal period on LATCH and breastfeeding self-efficacy scores.

**Method:**

A total of 80 pregnant who met the research criteria were randomly assigned to intervention (n = 40) or control (n = 40) groups. Pregnant women received to the control group received only standard care while breastfeeding education was accepted to the intervention group along with standard care. Both groups were visited at their home, and the personal data form, the LATCH Breastfeeding Assessment Tool, and Breastfeeding Self-Efficacy Scale–Short Form (BSES-SF) were applied in the postpartum 1st week. End of the study, brochures prepared by the researcher were given to both groups.

**Result:**

The mean breastfeeding self-efficacy and LATCH scores were higher in the intervention group compared to the control group. Breastfeeding success was found to increase as the maternal breastfeeding self-efficacy perception increased.

**Conclusion:**

Breastfeeding education given in the antenatal period increased maternal breastfeeding self-efficacy perception and breastfeeding success in the postpartum 1st week period.

Study is registered at ClinicalTrials.gov NCT04757324.

## RESUMO

**Objetivo:**

Inspecionar a influência da ajuda de treinamento em amamentação fornecida durante o processo pré-natal nos escores do LATCH e da autoeficácia da amamentação.

**Método:**

As gestantes, com número total de 80, que atendem aos critérios de investigação, foram arbitrariamente separadas em dois grupos, ou seja, um grupo de interferência (n = 40) e um grupo controle (n = 40). Enquanto as gestantes do grupo controle recebem apenas a cuidado normal, o grupo de interferência recebeu como extras os cuidados com a cuidado normal e o treinamento em amamentação. Ambos os grupos foram visitados em suas residências na primeira semana do puerpério e os formulários necessários à investigação, ou seja, o formulário de informações pessoais, LATCH Ferramenta de aproximação da amamentação e Formulários Curtos da Escala de Autoeficácia em Amamentação foram devidamente preenchidos. Etapa da investigação, foi entregue uma cartilha organizada pelo investigador para os dois grupos acima mencionados.

**Resultados:**

Comparado ao grupo controle, observou-se que o grupo interferência apresentou maiores médias de autoeficácia para amamentação e escores LATCH. Concluiu-se que quando se desenvolve a apreensão da mãe sobre a autoeficácia em amamentar, obviamente também se desenvolve a realização da amamentação.

**Conclusão:**

O treinamento em amamentação fornecido no período pré-natal desenvolveu a apreensão da autoeficácia da amamentação materna e a realização da amamentação na primeira semana do período pós-parto.

A investigação é registrada em ClinicalTrials.gov NCT04757324.

## RESUMEN

**Objetivo:**

Inspeccionar la influencia de la ayuda de capacitación en lactancia proporcionada durante el proceso prenatal en LATCH y las puntuaciones de autoeficacia en la lactancia.

**Metodo:**

Las gestantes, con un total de 80, que cumplieron con los criterios de investigación, fueron separadas arbitrariamente en dos grupos, es decir, un grupo de interferencia (n = 40) y un grupo de control (n = 40). Mientras que las mujeres embarazadas en el grupo de control solo reciben cura ordinaria, el grupo de interferencia recibió la cura ordinaria y capacitación en lactancia como extra. Ambos grupos fueron visitados en sus domicilios en la primera semana del puerperio y se cumplimentaron los formularios necesarios de la investigación, es decir, el formulario de datos personales, la Herramienta de Evaluación de Lactancia LATCH y los Formularios Breves de la Escala de Autoeficacia en la Lactancia Materna. Etapa de la investigación, se entregó un folleto organizado por el investigador a los dos grupos antes mencionados.

**Resultados:**

En comparación con el grupo de control, se observó que el grupo de interferencia tenía un promedio más alto de autoeficacia para amamantar y puntajes LATCH. Se concluyó que cuando se desarrolla la aprensión de la madre sobre la autoeficacia de amamantar, obviamente también se desarrolla el logro de amamantar.

**Conclusión:**

La capacitación en lactancia brindada en el período prenatal desarrolló la aprehensión de la autoeficacia de la lactancia materna y el logro de la lactancia materna en la primera semana del período posparto.

La investigación está registrada en ClinicalTrials.gov NCT04757324.

## INTRODUCTION

World Health Organization (WHO) recommends exclusive breastfeeding starting within one hour after birth until a baby is 6 months old. Nutritious complementary foods should be added while breastfeeding for up to 2 years or beyond^(1)^. The time to start breastfeeding is still far from the expectations world- wide despite the recommendations of the WHO. According to United Nations International Children’s Emergency Fund (UNICEF), the breastfeeding rate during the first 6 months of life did not change since 1990 and is around 36%^(2)^. The WHO reports that more than 820.000 children could be saved annually if breastfed until 2 years^(1)^.

Breastfeeding self-efficacy refers to a mother’s confidence in her capability to breastfeed her infant^(3)^. A high perception of maternal self-efficacy in breastfeeding is very effective for the maintenance of breastfeeding^(4)^. Studies indicate that early discontinuation of breastfeeding and early commencement of supplementary food result from breastfeeding-related problems. The worries about the discontinuation of breastfeeding and whether or not breast milk is sufficient for the baby may influence maternal self-efficacy in breastfeeding^(5)^. The mothers’ milk not being enough for her baby, concerns related to the mother’s milk not coming out, sore nipples, breast deformity, and anxiety about becoming a parent may also inﬂuence mothers’ breastfeeding self-efﬁcacy and breastfeeding capability^(6)^.

Practices for increasing maternal self-efficacy in breastfeeding are of vital importance, and they increase the level of maternal self-efficacy in breastfeeding^(7)^. Problems with the baby’s inability to latch properly in the postpartum period are common, contributing to breastfeeding cessation^(8)^. Breastfeeding success has been defined as a process that results in the mother and the baby’s satisfaction. Maternal self-efficacy in breastfeeding and correct breastfeeding techniques are essential for successful breastfeeding. Breastfeeding success is considered to increase as the maternal self-efficacy in breastfeeding perception increases^(7–9)^. LATCH score for assessment of breastfeeding practices has been widely used. Studies have shown that postpartum mothers with a LATCH score greater than 8 had higher breastfeeding success^(10)^.

Problems such as postpartum breast problems and giving the baby nutritional support and breastfeeding are experienced despite breastfeeding consultation provided in primary care, and these factors influence breastfeeding duration. Nurses should be aware of this and ensure that pregnant women receive proper and sufficient education in the antenatal period. The rate of exclusive breastfeeding is still much lower than the recommended rate, despite many studies worldwide ^(11)^. Antenatal breastfeeding education helps prepare women for effective breastfeeding by promoting their confidence level, knowledge, and skills. The authors hypothesized that the BSES-SF and LATCH scores of the women who receive a nursing education program would be higher than those who do not receive the nursing education program. This study aimed to examine the effects of a self-efficacy-based educational intervention on maternal breastfeeding self-efficacy and breastfeeding success in the postpartum 1^st^ week period.

## METHOD

### Type of Study

This was a two-group Quasi-Experimental study.

### Population

Pregnant women who applied to obstetric clinics were informed about the study. A list of pregnant women who wanted to participate in the study was created (n = 100). Pregnant women who did not meet the selection criteria were not included in the study (n = 20). Participants numbered from 1 to 80 using a computer program were divided into intervention (n = 40) and control (n = 40) groups by simple random sampling. Thirteen women from the intervention and control groups were excluded from the study. The study was completed with 67 (intervention n = 34, control n = 33) pregnant women.

### Local

The study was conducted in obstetrics clinics in Turkey between November 2016 and January 2018.

### Selection Criteria

Maternal selection criteria were mothers with no physical or mental illness, no medication for a particular disease, no structural defect in the breast, maternal willingness to breastfeed, over 18 years old, literate, no history of smoking, alcohol and drugs, no pregnancy complications. Newborn selection criteria were newborns with no congenital diseases or problems interfering with breastfeeding and no respiratory or cardiovascular issues requiring admission to the Newborn Intensive Care Unit (NICU).

### Data Collection

Our study took place in 3 stages. In stage 1: Contact details, estimated delivery date, and the written and verbal informed consents of the pregnant women in the intervention and control groups were obtained. Stage 2: Pregnant assigned to the control group (n = 40) received only standard antenatal and postnatal care. In Turkey, pregnant women are followed by the Ministry of Health eight times antenatal and once postpartum. Women assigned to the intervention group (n = 40) received breastfeeding education prepared for the breastfeeding self-efficacy theory developed by Dennis^(3)^ and standard antenatal and postnatal care. Maternal self-efficacy in breastfeeding refers to a mother’s confidence in her capability to breastfeed her infant^(3,12)^. The mother’s thinking that her milk is insufficient is due to her lack of self-confidence in her ability to breastfeed and cope with the difficulties during breastfeeding.

Educations were given in groups of 4–5 participants in the pregnant’s education room. It took about 4 hours in 2 sessions. Educations were given on weekdays when sufficient subjects were at the hospital. Verbal education, slides, models, video, and question and answer teaching methods were used. After the education, the intervention group was given the researcher’s phone number and told to call them when they had questions The mothers (n = 67) who constituted the study sample did not receive breastfeeding and breastfeeding training from a healthcare professional in the pregnant school/another institution. This prevented the response group from obtaining information from other sources.

The researcher giving education has a breastfeeding consultant certificate. In stage 3, Thirteen women from the intervention and control groups were excluded from the study. Six women in the intervention group, three women in the control group, withdrew from the study, and four in the control group newborns had been hospitalized at the intensive care unit; hence, these women were excluded from the sample Stage 3: The mothers in the intervention and control groups were visited at their, home and the personal data form, the LATCH Breastfeeding Assessment Tool and BSES-SF were applied in the postpartum 1^st^ week. End of the study, the control and intervention groups were given the same educational brochure prepared by the researcher.

Our training included the following topics; the importance of breast milk, breast problems, breast care, breastfeeding positions, breast rejection, milking and storing breast milk, breast-feeding of working mothers, milk sufficiency.

### Instruments

The data were collected using the “Personal Data Form,” the “Breastfeeding Self-Efficacy Scale-Short Form (BSES-SF),” and the “LATCH Breastfeeding Assessment Tool.”

### Personal Data Form

The researcher prepared the form following the literature and expert opinions ^(3,6,9,11,12)^. The form included questions about the mother’s age, education, working, gestational week, pregnancy planning, delivery type, parity, planning to breastfeed, first breastfeeding time, skin to skin contact and infant birth weight, infant gender, etc.

### Breastfeeding Self-Efficacy Scale-Short Form (BSES-SF)

The scale was first developed in 1999 as a 33 item scale. Some of the items were removed after that, and the 14 items short form was created, and the Cronbach’s alpha was found to be 0.94^(12)^. The scale is composed of 14 questions. It is a 5 Likert-type scale where 1 indicates “I am not sure” and 5 means “I am always sure.” The minimum score is 14, and the maximum score is 70. The higher scores indicate higher breast-feeding self-efficacy. The Cronbach’s alpha value was 0.86 in the Turkısh adaptation study^(11)^. The Cronbach’s alpha value was 0.72 in our study. BSES-SF scores range; from 14–32 are classified as low, from 33–51 medium, from 52–70 high self-efficacy perceptions^(13)^.

### LATCH Breastfeeding Assessment Tool

The scale was composed of the English initials of 5 assessment criteria as follows: L (Latch on the breast), A (Audible swallowing), T (Type of the nipple), C (Comfort breast/nipple), and H (Hold). Each item is scored between 0 and 2, and the maximum score is 10^(14)^. The breastfeeding success increases as the score increases. Turkish reliability and validity study of the scale and determined the Cronbach’s alpha value is 0.95^(9)^. The Cronbach’s alpha value was 0.78 in our study.

### Ethical Aspects

All study participants provided informed consent, and the appropriate ethics review board approved the study design. Before conducting the study, ethical approval was obtained from the local state hospital Clinical Research Ethics Committee in October 2016. (decision number: 2016/100-decision date: 19.10.2016). Permission for the study was also received from the hospital. The Clinical Trials registration number was NCT04757324 https://clinicaltrials.gov/ct2/show/NCT04757324?term=NCT04757324&draw=2&rank=1.

### Data Analysis and Treatment

The data were analyzed using the SPSS 22.0 package program (The Statistical Package for the Social Sciences). Descriptive statistics included means and standard deviations (SD) for continuous variables. Frequencies and percentages were used for categorical variables for demographic and breastfeeding characteristics of the intervention and control groups at baseline. Kolmogorov-Smirnov test was used to evaluate the suitability of the data for normal distribution, and data did not have a normal distribution, so non-parametric tests were used. To compare differences in characteristics between the intervention and control groups, the chi-square test, Fisher’s exact test, and chi-square test with the Monte Carlo simulation helps were performed. Mann-Whitney U test was used to compare the 1^st^ week postpartum BSES-SF scores and LATCH scores between the groups. Pearson correlation analysis was used to measure the relationship between BSES-SF and LATCH scores. The Cronbach’s alpha reliability coefficients of the scales were determined using reliability analysis. In addition, a power analysis was performed to reveal the power of the study. The results were evaluated at a confidence interval of 95%, and the significance level was established at p < 0.05.

## RESULTS

**Figure 1.**
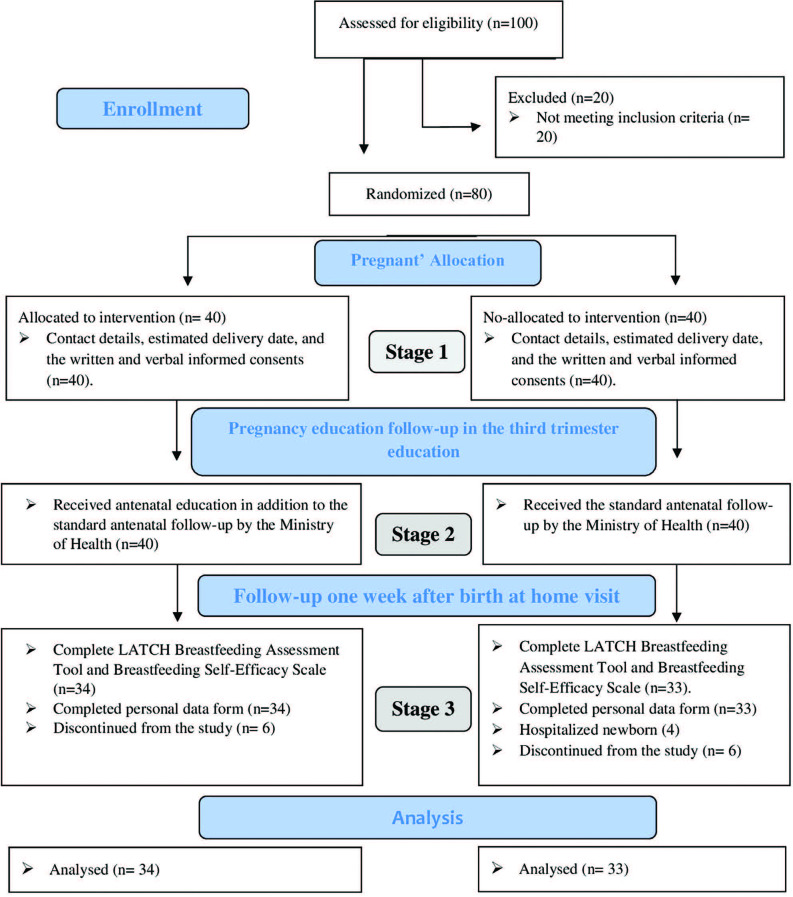
CONSORT flow diagram of this study; adapted according to Consort (http://www.consort-statement.org/consort-statement/flow-diagram).

The effect of breastfeeding education given in the antenatal period on maternal breastfeeding self-efficacy and breastfeeding success were evaluated in the postpartum 1^st^ week. Of the 100 women who participated in the survey, 80 of them met the sampling criteria of the mother and were included in the study. Thirteen women from the intervention and control groups were excluded from the study. Six women in the intervention group and three women in the control group withdrew from the study. Four in the control group newborns had been hospitalized at the intensive care unit; hence, these women were excluded from the sample. The study was completed with 67 mothers and newborns (intervention group = 34, control group n = 33) (Figure 1). Baseline information of the intervention and control groups participants was checked before analyzing the impact of antenatal education. The analysis identified that both control and intervention groups were similar in age, educational status, gestational age, working status, planning of pregnancy, type of delivery, parity, infant gender (Table 1). A statistically significant difference was detected in favour of the intervention group about the planned breastfeeding duration (p = 0.001) and the skin-to-skin contact just after birth (p = 0.001) (Table 2).

**Table 1.** Comparison of mothers’ baseline information – Balıkesir, Marmara Region, Turkey, 2018.

	Intervention		Control		Total		χ^2^	P
	n	%	n	%	n	%		
**Age**								
18–25	15	44.1	15	45.4	30	44.8	0.23	0.89
26–30	14	41.2	12	36.4	26	38.8		
31+	5	14.7	6	18.2	11	16.4		
**Educational status**								
Primary education	0	0	3	9.1	3	4.5	1.79	0.18
High school	13	38.2	15	45.5	28	41.8		
University	21	61.8	15	45.4	36	53.7		
**Working status**								
Employed	14	41.2	6	18.2	20	29.8	3.20	0.07
Unemployed	20	58.8	27	81.8	47	70.2		
**Planning of pregnancy**								
Planned	27	79.4	27	81.8	54	80.6		1.00^a^
Not planned	7	20.6	6	18.2	13	19.4		
**Type of delivery**								
Vaginal	19	55.9	16	48.5	35	52.2	0.13	0.71
Cesarean section	15	44.1	17	51.5	32	47.8		
**Parity**								
Primiparous	27	79.4	25	75.8	52	77.6	0.004	0.94
Multiparous	7	20.6	8	24.2	15	22.4		
**Gestational week**								
38 week	0	0	1	3.0	1	1.5		1.00^b^
39 week	5	14.7	4	12.1	9	13.5		
40 week	28	82.3	27	81.8	55	82.1		
41-42 week	1	3.0	1	3.1	2	2.9		
**Infant gender**								
Male	17	50.0	13	39.3	30	44.8	0.39	0.53
Female	17	50.0	20	60.7	37	55.2		
**Total**	**34**	**100**	**33**	**100**	**67**	**100**		

χ^2^ = Chi-Square Test; ^a^Fisher’s Exact Test; ^b^Chi-Square Test with The Help of Monte Carlo Simulation.

**Table 2.** Comparison of Descriptive Characteristics of Breastfeeding – Balıkesir, Marmara Region, Turkey, 2018.

Breastfeeding characteristics	Intervention		Control		Total		χ^2^	P
	n	%	n	%	n	%		
**Immediately after birth**								
Within first 30 minutes	16	47.0	7	21.2	23	34.3	5.34	0.06
30–60 minutes	14	41.2	18	54.5	32	47.8		
61 minutes and above	4	11.8	8	24.2	12	17.9		
**Planned breastfeeding period**								
Within first 6 months	4	11.8	16	48.5	20	29.9	16.6	0.001
6–12 months	3	8.8	7	21.2	10	14.9		
12–24 months	27	79.4	10	30.3	37	55.2		
**Skin-to-skin contact**								
Yes	12	35.3	1	3.0	13	19.4	11.14	0.001
No	22	64.7	32	97.0	54	80.6		
**Total**	**34**	**100**	**33**	**100**	**67**	**100**		

χ^2^ = Chi-Square Test.

When the mothers’ BSES-SF scores were compared, the mean score was 61.12 ± 4.06 in the intervention group and 58.39 ± 5.17 in the control group in the postpartum 1st week period. The mothers’ BSES-SF scores in the intervention group were significantly higher than those in the control group, and the difference was statistically significant (p = 0.03). The mean LATCH score was 8.38 ± 1.50 in the intervention group and 7.30 ± 1.51 in the control group. The mean LATCH scores of the mothers who received breast milk and breastfeeding education were higher than those in the control group, and the difference was statistically significant (p = 0.003) (Table 3). A positive correlation was determined between the mean BSES-SF scores and the LATCH scores in the intervention and the control groups (p = 0.003). The LATCH scores increased as the BSES-SF scores increased (r = 0.345) (Table 4).

**Table 3.** Comparison of The 1^st^ Week Postpartum Breastfeeding Self Efficacy and LATCH Scores Between Groups – Balıkesir, Marmara Region, Turkey, 2018.

	n	Mean	Median	Min	Max	SD	Mean rank	z	P
**Breastfeeding self-efficacy (BSES-SF) score**									
Intervention	34	61.12	62.0	50.0	68.0	4.06	39.21	−2.2	0.03
Control	33	58.39	59.0	48.0	67.0	5.17	28.64		
**Total**	**67**	**59.78**	**61.0**	**48.0**	**68.0**	**4.80**			
**LATCH breastfeeding assessment tool score**									
Intervention	34	8.38	9.0	5.0	10.0	1.50	40.78	−3	0.003
Control	33	7.30	7.0	4.0	10.0	1.51	27.02		
**Total**	**67**	**7.85**	**8.0**	**4.0**	**10.0**	**1.59**			

Mann- Whitney U Test.

**Table 4.** Correlation Between Breastfeeding Self-Efficacy (BSES-SF) and LATCH Breastfeeding Assessment Tool Scores – Balıkesir, Marmara Region, Turkey, 2018.

LATCH breastfeeding assessment tool scores		
Breastfeeding Self-Efficacy (BSES-SF) Scores	r	0.345
	p	0.004
	**n**	**67**

Pearson correlation analysis.

## DISCUSSION

The World Health Organization recommends breastfeeding within the first hour after delivery. Globally, 44% of newborns breastfeed within the first hour after birth, and 42% of infants under 6 months of age are exclusively breastfed^(15)^. In our study, while 47.1% of the mothers in the intervention group breastfed their babies within the first 30 min after delivery, this rate was 21.1% in the control group. In the present study, the mothers in the intervention group could breastfeed their babies 2.2 fold earlier than the mothers in the control group.

BSES-SF scores range; from 14–32 are classified as low, from 33–51 medium, from 52–70 high self-efficacy perceptions^(13)^. In our study, both groups’ perception of maternal breastfeeding self-efficacy was also found to be high, still, mothers in the intervention group (61.12 ± 4.06) had a higher when compared to the control group (58.39 ± 5.17) (p = 0.03). We thought that the high levels of maternal breastfeeding self-efficacy in both groups is that health services are provided free of charge in Turkey and that every woman has reached standard antenatal and postnatal care.

Interventions focusing on four sources (performance accomplishment, vicarious experiences, verbal persuasion, and physiological responses) of breastfeeding self-efficacy can eventually increase breastfeeding self-efficacy^(11–13,16–19)^. Mothers who can latch the babies and be guided to handle breastfeeding difficulties during the antenatal can achieve performance accomplishment, and vicarious experience (peers, friends, etc.) can perform better^(13,16–19)^. İt has been stated in the literature that breastfeeding self-efficacy theory-based educational interventions nursing interventions in the antenatal period increase maternal breastfeeding self-efficacy in the postpartum period, significant differences between the intervention and control groups^(17,18,19,20)^. Researchers randomized 240 women into two groups in a study conducted in Brazil. Telephone education intervention was applied to the intervention group at 7, 30, 90, and 150 days after birth. Women in the education intervention group had higher perceptions of maternal breastfeeding self-efficacy when compared to the control group^(13)^. These results confirm that interventions developed with breastfeeding self-efficacy theory can significantly increase breastfeeding self-efficacy.

The WHO recommends skin-to-skin contact between the mother and the baby after delivery and encourages mothers to breastfeed and help them for breastfeeding^(21)^. Iranian researchers found that the BSES-SF scores, the breastfeeding initiation rates, and the time to start the first breastfeeding were statistically significantly better in mothers who had skin-to-kin contact with their babies^(5)^. In this study, 35.3% of the mothers in the intervention group and 3.0% of the mothers in the control group had skin-to-skin contact just after delivery. The skin-to-skin contact rate of the babies in the intervention group was statistically significantly higher.

Studies have shown that postpartum mothers with a LATCH score greater than 8 had higher breastfeeding success^(6,8,10)^. In our research, we found the mean LATCH score of the mothers to be 8.38 ± 1.50 and; LATCH scores in the intervention group were higher than those in the control group, and the difference was statistically significant. Many studies in the literature report that breastfeeding education is given in the antenatal period and the early postpartum period increases maternal breastfeeding self-efficacy and success^(11–12,19,22,23,24)^. Therefore, nurses’ antenatal breastfeeding support and education play a crucial role in breastfeeding self-efﬁcacy, breastfeeding capability, and the mother’s decision to initiate and continue breastfeeding^(25)^. Other studies indicated that the mothers’ BSES-SF scores and LATCH scores in the intervention group were significantly higher than those in the control group^(11,26–27)^. In an intervention study in India, lactating mothers with low LATCH scores at the initial evaluation, an accurate breastfeeding technique were recommended by a lactation nurse to improve the score before discharge, and a correct breastfeeding technique was demonstrated; when it was valued in the 6^th^ week, they found that the LATCH score increased^(8)^.

Our study analysed the correlation between the BSES-SF scores and the LATCH scores of the intervention and the control groups. The BSES-SF scores were found to increase as the breastfeeding success increased, and a positive correlation was found between them. Our study results are consistent with the studies in the literature investigating maternal breastfeeding self-efficacy and breastfeeding success^(6,28–29)^.

## Limitations of the Study

Giving educations to groups of 4–5 subjects is a time-consuming practice despite being effective. The effectiveness of the educations given to larger groups is not known. The results obtained at the postpartum the 1^st^ week have been presented in the study; however, the maternal breastfeeding self-efficacy and the breastfeeding success were not evaluated later. The difficulty in its application in the field and the absence of the data in the later period are limitations of the present study.

## CONCLUSION

In conclusion, breastfeeding education in the ante-natal period positively influences maternal breastfeeding self-efficacy and breastfeeding success. According to this result, education and consultations for breastfeeding self-efficacy and success are recommended for mothers starting from the antenatal period.

## ASSOCIATE EDITOR

Ivone Evangelista Cabral

## References

[1] World Health Organization (2016). World breastfeeding week [Internet].

[2] United Nations International Children’s Emergency Fun (2016). Breastfeeding on the World wide agenda [Internet].

[3] Dennis CL (1999). Theoretical underpinnings of breastfeeding conﬁdence: A
self-efﬁcacy framework. J Hum Lact.

[4] Joshi A, Trout KE, Aguirre T, Wilhelm S (2014). Exploration of factors influencing initiation and continuation of
breastfeeding among Hispanic women living in rural settings: A multi-methods
study. Rural Remote Health.

[5] Azami-Aghdash S, Ghojazadeh M, Dehdılanı N, Mohammadı M (2014). Prevalence and causes of cesarean section in Iran: Systematic
review and meta-analysis. Iran J Public Health.

[6] Gerçek E, Sarikaya KS, Ardic CN, Saruhan A (2017). The relationship between breastfeeding self-efficacy and LATCH
scores and affecting factors. J Clin Nurs.

[7] Gökçeoğlu E, Küçükoğlu S (2017). The relationship between insufficient milk perception and
breastfeeding self-efficacy among Turkish mothers. Global Health Promotion.

[8] Shah MH, Roshan R, Parikh T, Sathe S, Vaidya U, Pandit A (2021). LATCH score at discharge: a predictor of weight gain and
exclusive breastfeeding at 6 weeks in term healthy babies. J Pediatr Gastroenterol Nutr.

[9] Yenal K, Okumuş H (2003). Reliability of LATCH breastfeeding assessment
tool. HEMAR-G.

[10] Buranawongtrakoon S, Puapornpong P (2016). Comparison of latch scores at the second day postpartum between
mothers with cesarean sections and those with normal
deliveries. Thai Journal of Obstetrics and Gynaecology.

[11] Tokat MA, Okumuş H (2013). Mothers breastfeeding self-efficacy and success: analysis of the
effect of education based on ımproving breastfeeding
self-efficacy. HEMAR-G.

[12] Dennis CL, Faux S (1999). Development and psychometric testing of the breastfeeding
self-efﬁcacy scale. Res Nurs Health.

[13] Dodou HD, Bezerra RA, Chaves AFL, Vasconcelos CTM, Barbosa LP, Oriá MOB (2021). Telephone intervention to promote maternal breastfeeding
self-efficacy: randomized clinical trial. Rev Esc Enferm USP.

[14] Jensen D, Wallace S, Kelsay P (1994). LATCH: A breastfeeding charting system and documentation
tool. J Obstet Gynecol Neonatal Nurs.

[15] United Nations International Children’s Emergency Fund. The State of
the World’s Children (2019). Children, Food and Nutrition: Growing well in a changing world
[Internet].

[16] Imdad A, Yakoob MY, Bhutta ZA (2011). Effect of breastfeeding promotion interventions on breastfeeding
rates, with special focus on developing countries. BMC Public Health.

[17] Araban M, Karimian Z, Kakolaki ZK, McQueen KA, Dennis CL (2018). Randomized controlled trial of a prenatal breastfeeding
self-efficacy intervention in primiparous women in Iran. J Obstet Gynecol Neonatal Nurs.

[18] Çitak Bilgin N, Ak B, Ayhan F, Kocyigit F, Yorgun S, Topcuoglu MA (2020). Effect of childbirth education on the perceptions of childbirth
and breastfeeding self efficacy and the obstetric outcomes of nulliparous
women. Health Care Women Int.

[19] McQueen KA, Dennis CL, Stremler R, Norman CD (2011). A pilot randomized controlled trial of a breastfeeding
self-efficacy ıntervention with primiparous mothers. J Obstet Gynecol Neonatal Nurs.

[20] Mızrak B, Özdoıan N, Çolak E (2017). The effect of antenatal education on breastfeeding self-efficacy:
Primiparous women in Turkey. International Journal of Caring Sciences [Internet].

[21] World Health Organization (2013). Essential nutrition actions: improving maternal, newborn, infant and
young child health and nutrition [Internet].

[22] Perez-Blasco J, Viguer P, Rodrigo MF (2013). Effects of a mindfulness-based intervention on psychological
distress, wellbeing, and maternal self-efﬁcacy in breast-feeding mothers:
Results of a pilot study. Arch Womens Ment Health.

[23] Rojjanasrirat W, Nelson EL, Wambach KA (2012). A pilot study of home-based videoconferencing for breast-feeding
support. J Hum Lact.

[24] Wu DS, Hu J, McCoy TP, Efird JT (2014). The effects of a breastfeeding self-efficacy ıntervention on
short-term breastfeeding outcomes among primiparous mothers ın Wuhan,
China. J Adv Nurs.

[25] Fu I, Fong D, Heys M, Lee I, Sham A, Tarrant M (2014). Professional breastfeeding support for first-time mothers: A
multicentre cluster randomized controlled trial. BJOG.

[26] Brockway M, Benzies K, Hayden KA (2017). I˙ nterventions to improve breastfeeeding self-efficacy and
resultant breastfeeding rates: A systematic review and
meta-analysis. J Hum Lact.

[27] Lee YH, Chang GL, Chang HY (2019). Effects of education and supports groups organized by IBCLCs in
early postpartum on breastfeeding. Midwifery.

[28] İnce T, Aktaş G, Aktepe N, Aydın A (2017). Evaluation of the factors affecting mothers’ breastfeeding
self-efficacy and breastfeeding success. Izmir Dr. Behçet Uz Çocuk Hastanesi dergisi.

[29] Yenal K, Tokat MA, Ozan Durgun Y, Çeçe Ö, Abalin FB (2013). The relation between breastfeeding self-efficacy and
breastfeeding successes of mothers. HEMAR-G [Internet].

